# Estimating Motorcycle Telescopic Fork Suspension Travel Speed with Four-Degree-of-Freedom Full-Vehicle Kalman Filter

**DOI:** 10.3390/s26103029

**Published:** 2026-05-11

**Authors:** Alberto Ponso, Saulius Pakštys, Angelo Bonfitto

**Affiliations:** Department of Mechanical and Aerospace Engineering, Politecnico di Torino, Corso Duca degli Abruzzi 24, 10129 Turin, Italy; alberto.ponso@polito.it (A.P.); angelo.bonfitto@polito.it (A.B.)

**Keywords:** Kalman filter, elongation speed estimation, motorcycle in-plane dynamics, suspension dynamics, vehicle modeling

## Abstract

This paper concerns the estimation of front telescopic fork suspension elongation speed through the use of Kalman-filtering techniques. A full-motorcycle model in the state-space domain is developed and subsequently used in the filter along with synthetic input data simulating two accelerometer measurements. In addition, the force of semi-active suspension is considered as an input, from which the value is estimated on the basis of a look-up table and the estimated elongation speed. The performance of the full-motorcycle filter is compared to that of a filter built considering the monocorner model, indicating superiority in performance. The ratio of the mean squared error of the suspension elongation speed to the mean square of the elongation speed originating from the non-linear model is used as a performance metric. For the proposed estimator, it is 6.54% with respect to the best class of road profile (A) and 7.07% for the worst (H). This is in contrast to the monocorner filter, displaying values of 57.46% and 94.47% for the best and worst road classes, respectively. The influence of system pitch dynamics is evidenced to have a marginal influence on the accuracy of speed estimation. However, it is the use of a larger set of states that adds the notable advantage of employing such a solution.

## 1. Introduction

With road transport continuing to grow at a rapid pace, the requirement for solutions in the domain of control and comfort is ever increasing. Indeed, global vehicle sales rose from 66.2 to 77.6 million between the years 2022 and 2025 [1], with over 58 million electric vehicles being present on the road in 2025 [2]. The general trend indicates a rise in demand for vehicles and personal mobility, including two-wheelers such as motorcycles. A variety of sub-systems affect the dynamics and stability of a vehicle, which include, but are not limited to, brakes, steering, the powertrain and suspensions. This last sub-system has drawn significant attention in the literature, as it is composed of a key group of components that play a significant role in vehicle dynamics. Suspensions serve the purpose of isolating the vehicle body from vibrations transmitted from road irregularities and may be realized through passive, semi-active and fully active types [3]. Semi-active suspension systems have drawn attention in regard to their development, as they offer the best compromise between application cost and performance. The literature is generally divided into two paths: (1) development of new technologies such as electrohydraulic and magneto-rheological systems and (2) design of active control strategies [4]. These streams of development are also evident in the design and control of suspension systems for motorcycles, which have the same purposes as those of four-wheeled vehicles. The sub-systems take the form of different topologies when considering the front and rear of the motorcycle. With regard to the front, the most popular suspension type is the telescopic fork, in which two telescopic sliders produce a prismatic joint between the unsprung and sprung masses [5]. It is relatively simple to implement and shows low inertia around its axis of rotation. The limitations of these suspension types are the presence of dry friction, which is typically associated with prismatic joints, as well as higher values of unsprung mass compared to other solutions. To counteract these limitations, alternative mechanical designs have been proposed, such as the push-arm, trail-arm and four-bar linkage [5,6]. The work herein focuses on the telescopic fork, as it is the most widely adopted suspension type. The rear suspension, on the other hand, is typically designed through a swing arm composed of two oscillating arms and two mass-damper elements found on each side. It is relatively simple in its construction but has a limitation: poor force–displacement characteristics. The cantilever mono-shock system and the four-bar linkage are alternative solutions developed to overcome the limitations of torsional stress generation on the swing arm and the poor progressive force–displacement characteristic it displays. These designs are not evaluated in this work, as the focus is not on their optimal design but rather the effect the rear suspension has on the estimation of the front suspension system’s travel speed.

To properly damp the system through active control, an adequate set of feedback variables is necessary, among which the elongation velocity between the unsprung and sprung masses is of fundamental importance. The precision of this variable is crucial to perform suspension control effectively, given the significant non-linear behavior of this sub-system [7]. The value of the velocity can typically be obtained by using linear potentiometers or accelerometers. The former are known to provide an accurate measurement; however, they are subject to high impact forces that cause damage and failure [8]. The latter are mainly affected by measurement noise, an issue that has motivated multiple studies to investigate filter-based observers [9,10,11,12]. In a study by Delvecchio et al. [13], an implementable force estimator is developed for a motorcycle semi-active suspension, in which two observers of the elongation speed are developed based on Kalman-filtering techniques, proposed by Rudolph E. Kalman [14]. The first relies on the accelerations measured by one sensor, while the second considers the use of two sensors. Regardless of the number of accelerometers employed, model-based estimation of the elongation speed is necessary because direct integration of the difference of the two measurements is not feasible due to the presence of noise. The same authors also propose the estimation of vertical tire deflection using Kalman-filtering techniques, citing a necessity due to the high cost and infeasibility of solutions based on optical sensors [15]. Both studies were carried out considering a monocorner model, i.e., the implementation of a system of equations equivalent to a single quarter car model, the most widespread representation to analyze tire–suspension dynamics.

The accuracy level such a model provides is adequate when considering low amplitudes of vertical road speed as disturbance inputs to the system. However, as the amplitudes increase, the effect of the rear unsprung mass has a greater impact on the estimation of the front suspension travel speed. In addition, it only considers pure vertical dynamics (heave) and the influence of pitch dynamics is not captured. While the decoupling of heave and pitch is the standard in passenger car modeling [16], their interaction may become significant for the comfort of the rider and road-holding of a motorcycle, where the related natural frequencies differ [5]. In particular, frequencies over 2 Hz are common for the heave motion of the sprung mass, which is higher than the corresponding value (∼1 Hz) in passenger cars [17,18]. Consequently, vibrational analyses involving motorcycles consider both wheels together, due to the influence of pitch angles on suspension travels [18,19]. This requires the use of a higher-order model, considering the full motorcycle as shown in the work of Yuan et al. [20]. The study analyzed the derivation of the state equations for a five-degree-of-freedom (DOF) system and evaluated the model’s performance considering a road disturbance. However, it does not consider the estimation of the elongation speed of the telescopic suspension. In the work of Segla and Roy [21], the authors model the motorcycle as a viscoelastically suspended rigid body with four degrees of freedom for the purpose of comfort evaluation when the vehicle is subjected to a road disturbance in the form of a bump. The study does not consider the use of the model to estimate telescopic speed.

To the best of the authors’ knowledge, a higher-order model filtering technique for estimation of the elongation travel speed of a motorcycle telescopic fork suspension has not been addressed by the research field. Therefore, the present work discusses the estimation of the variable through the implementation of a Kalman filter based on a full motorcycle model, presenting an in-depth analysis of the effects of higher model fidelity on variable estimation. Two acceleration measurements are used as model inputs, along with the force of the semi-active damper, which is estimated by implementing an appropriate look-up table. The work provides a performance evaluation of the full model filter with that of a Kalman filter based on a monocorner model. Comparisons between the two filter versions show an improvement in the mean squared error, which can be achieved with the increase in model complexity, which reduces by 92.5% in the worst road profile class. In addition, the effect of the motorcycle pitch inertia on the suspension is considered with its acceleration and deceleration following the World Motorcycle Test Procedure Cycle (WMTC). The remainder of the paper is structured as follows. First, the development methodology of the two filters is discussed, followed by the modeling of the system through a state-space representation. The results and discussion considering synthetic data are presented afterwards, with the final remarks and future work concluding the article.

## 2. Methodology

The main purpose of a Kalman filter is to reconstruct a non-measurable state of a system starting from command inputs and measured outputs. To do so, the equations of the system must be defined in state-space form [14]. The state-space representation of a system is written as a combination of two functions, defining the derivative of the state variable vector and the measured output vector as functions of the current state vector and of the input commands [22]. Additionally, the state transition function *f* is affected by input disturbances and the output is corrupted by noise on measurement instruments [23]. A generic representation of a non-linear state-space is presented in (1):

(1) $$\left\{\begin{array}{c} \dot{x}=f\left(x,u,w\right)\\ y=h(x,u,w,v) \end{array}\right.$$

where *x* represents the state vector, *u* is the command input, *w* is the disturbance input and *v* is the noise affecting the measurement of the output *y*. Disturbances *w* and *v* are characterized by covariance matrices *Q* and *R*, respectively, and are uncorrelated. Therefore, their noise cross-covariance matrix *N* is identically nil.

However, in control theory, great interest is given to linear systems where the relation between state-space, its derivative, input commands, and output measurement is given by matrices [24]. The definition of such a system is shown in (2):

(2) $$\left\{\begin{array}{c} \dot{x}=Ax+Bu+Gw\\ y=Cx+Du+Hw+v \end{array}\right.$$

where A is the state-transition matrix, B is the input gain matrix, G is the input gain matrix for disturbances, C is the output gain matrix, D the feed-through matrix for command inputs, and H is the feed-through matrix for disturbances. The expression is written for the case of continuous systems; however, discretization in time is required such that it can be implemented for real-time applications. To discretize the state-space representation, the input quantities *u* are assumed to be constant during a sampling instant $t_{k}=T_{s}$ and the disturbance input gain matrix is neglected. Thus, the following expression can be derived:

(3) $$\begin{array}{cc} \; & x_{k}-x_{k-1}=AT_{s}x_{k-1}+BT_{s}u_{k},\\ \; & x_{k}=\left(I+AT_{s}\right)x_{k-1}+BT_{s}u_{k-1} \end{array}$$

where *k* is the discrete time instant, the expression $\left(I+AT_{s}\right)$ is termed as the transition matrix Φ, and the expression $BT_{s}$ is the discrete time input matrix F.

Considering Kalman filter applications, the method concerns itself with linear systems, and represents beliefs (predictions) by the moments in which, at a given time *t*, the belief is represented by the mean (state prediction) ${\bar{x}}_{k}$ and the covariance $P_{k}$ [25]. With reference to expressions (2) and (3), the expression for the state transition can be written as follows:

(4) $$\begin{array}{c} x_{k}=\Phi_{k}x_{k-1}+F_{k}u_{k}+w_{k} \end{array}$$

in which $w_{k}$ is the process uncertainty having zero mean and covariance matrix $Q_{k}$. It is a Gaussian random vector modeling unpredictability in the state transition. The measurement, on the other hand, is expressed as follows:

(5) $$\begin{array}{c} z_{k}=H_{k}x_{k}+v_{k} \end{array}$$

with $v_{k}$ being the vector of measurement uncertainty having zero mean and covariance matrix $R_{k}$. The prediction of the state vector $\bar{x}$ of the system makes use of the discrete state-space system, along with the covariance matrix $P_{k}$:

(6) $$\begin{array}{cc} \; & {\bar{x}}_{k}=\Phi_{k}x_{k-1}+F_{k}u_{k} \end{array}$$

(7) $$\begin{array}{cc} \; & {\bar{P}}_{k}=\Phi_{k}P_{k-1}\Phi_{k}^{⊤}+Q_{k}. \end{array}$$

The correction of the predicted value is performed with the use of the Kalman gain matrix $K_{k}$:

(8) $$\begin{array}{c} K_{k}={\bar{P}}_{k}{F_{k}}^{⊤}{\left(H_{k}{\bar{P}}_{k}^{⊤}+R_{k}\right)}^{-1} \end{array}$$

and is subsequently implemented in the following formulation to obtain the correct state vector:

(9) $$\begin{array}{c} x_{k}={\bar{x}}_{k}+K_{k}\left(z_{k}-H_{k}{\bar{x}}_{k}\right). \end{array}$$

The covariance matrix is then updated through:

(10) $$\begin{array}{c} P_{k}=\left(I-K_{k}H_{k}\right){\bar{P}}_{k}. \end{array}$$

Instead of the two-step prediction-correction process shown in expressions (6)–(10), an alternative way to define $K_{k}$ in a single step exists and it was demonstrated that the two methods exhibit the same convergence properties [26]. The update of the covariance matrix P and the calculation of the gain matrix $K_{k}$ [27] are:

(11) $$\begin{array}{cc} \; & P_{k+1}=A_{k}P_{k}{A_{k}}^{⊤}+Q_{k}-K_{k}\left(C_{k}P_{k}{C_{k}}^{⊤}+R_{k}\right)K_{k} \end{array}$$

(12) $$\begin{array}{cc} \; & K_{k}=A_{k}P_{k}{C_{k}}^{⊤}{\left(C_{k}P_{k}{C_{k}}^{⊤}+R_{k}\right)}^{-1}. \end{array}$$

It has been demonstrated that the matrix of the estimation error covariance $P_{k}$ converges to a steady state constant value ${\bar{P}}_{k}$ [28]. This implies that if the sensing noise covariance $R_{k}$ is constant, the gain matrix $K_{k}$ converges to a steady state value as well. In the case where process noise *w* is additive and matrices G and H are known, formulation of (12) is further modified to account for their impact [29]:

(13) $$\begin{array}{cc} K_{k}= & \left(A_{k}P_{k}{C_{k}}^{⊤}+G_{k}Q_{k}{H_{k}}^{⊤}\right)\cdot \\ & {\left(C_{k}P_{k}{C_{k}}^{⊤}+R_{k}+H_{k}Q_{k}{H_{k}}^{⊤}\right)}^{-1} \end{array}$$

This formulation is preferred over the previous two-step prediction-correction method, due to its single-step procedure, and is therefore exploited in this work.

**Figure 1:**
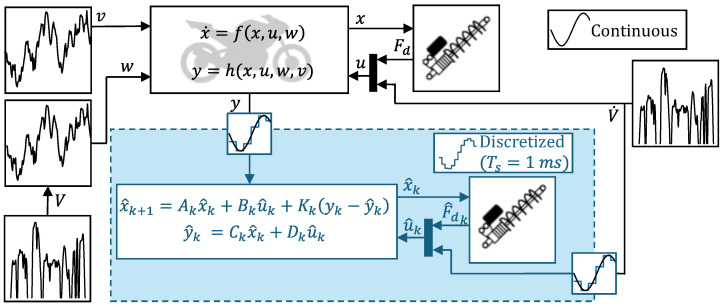
Layout of simulation of motorcycle running on random road profile. Blocks in blue represent discrete state space.

Modeling and simulation of the motorcycle with parallel state estimation are performed in Matlab&Simulink^®^ (R2024b) following the scheme represented in Figure 1.

**Figure 2:**
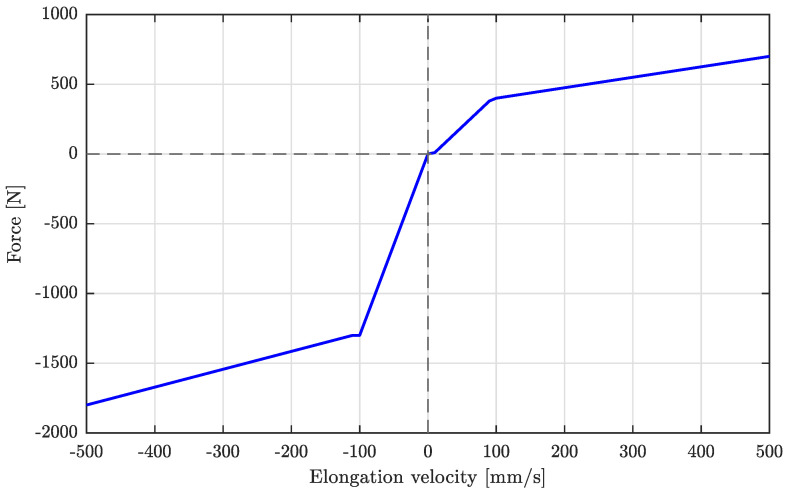
Force against elongation velocity characteristic of the damper employed for a zero current command, taken from [13].

Even though the vehicle model is non-linear, the employed Kalman filter is based on a linear time-invariant (LTI) system, following the examples present in the literature [13,30]. This approximation is made possible by the relatively small pitch angles, whose root mean square (RMS) is less than 0.15 rad for all considered road profiles. Command inputs to the model are the force exerted by a semi-active damper and the longitudinal acceleration, while the disturbance input is represented by the road profile vertical displacement. Although sensors that measure the force applied by hydraulic dampers exist [31], the value of $F_{d}$ is not always directly measurable, either due to the type of suspension installed or to reduce the number of mounted sensors. Therefore, to be able to deal with systems where only accelerometers are installed, the state estimation of Figure 1 also includes a damper model, estimating the force ${\hat{F}}_{d_{k}}$ applied at time step *k*. As the force–speed characteristic is highly non-linear, it is typically implemented through experimental look-up tables (LUTs) [13]. In this work, the LUT is taken from [13] where a semi-active damper is characterized under the actuation of different electrical currents. As the damper employed in this work is passive, the curve for 0 mA is used, shown in Figure 2. As the LUT computes ${\hat{F}}_{d_{k}}$ based on the estimated elongation speed, small errors in the estimation of one of the two can propagate and lead to significant errors. One of the solutions when dealing with uncertain inputs is state augmentation, i.e., the creation of a larger state space including said inputs as elements of the state vector or by including unknown input biases in the state vector instead [32,33]. However, as ${\hat{F}}_{d_{k}}$ can be considered as dependent only on the elongation speed for passive dampers, its computation in the literature is generally performed from the estimated state ${\hat{x}}_{k}$ as in Figure 1 [13,30].

The non-linear vehicle model receives the true commands *u* as inputs, while the linearized Kalman filter receives the estimated commands, $\hat{u}$. Noise-affected measurements are relayed to the Kalman filter, which utilizes them to reconstruct $\hat{x}$. With reference to previous applications that consider the estimation of a motorcycle front fork travel speed, two outputs are measured: vertical acceleration of the front unsprung mass and of the sprung mass [13]. Particularly, it is assumed that the worst affected measurement is the latter, due to higher sensitivity to engine vibrations. The noise level associated with the two measurements in this study is informed by [13], where values of up to 1 g are seen for the sprung mass and 0.5 g for the unsprung mass. In this work, noise values of 0.3 g on the sprung and 0.1 g on the unsprung masses are imposed, considering the study of [34] that indicates values of up to 0.15 g evident for industrial accelerometers within an excitation frequency range of 1–15 Hz. The output of the filter is the estimated state $\hat{x}$, fed into a higher-level control which can be used to adjust semi-active damper settings to ensure the desired level of comfort and handling performance.

To evaluate the performance of the Kalman filter in tracking the value of fork elongation speed, motorcycle in-plane dynamics during straight-line travel of the motorcycle are simulated, a procedure that is commonly used in the design and testing of suspension sub-systems [35,36]. A random road profile is generated according to ISO Standard 8608 [37]. It provides a disturbance input to the vehicle model as vertical displacement in the form of vector *w*. Since the study analyzes vehicle longitudinal motion, rear wheel displacement $z_{g,r}$ is obtained by delaying the front wheel displacement $z_{g,f}$ by a number of time steps *n* depending on speed *V*, wheelbase *l* and sample time $T_{s}$ as in:

(14) $$\begin{array}{c} n=\left\lceil \frac{l}{VT_{s}}\right\rceil . \end{array}$$

Generation of the vertical displacement input is conducted by filtering white noise with a low-pass transfer function, as indicated in [38]:

(15) $$\begin{array}{c} H\left(s\right)=\frac{2\pi \sqrt{G_{v}\cdot V}}{s+\omega_{0}}. \end{array}$$

In (15), $G_{v}$ represents the road roughness index, while $\omega_{0}$ is the cut-off pulsation, imposed as 1.22 rad/s, from the previous application of such a technique in the validation performance of shock absorbers [39]. The value of $G_{v}$ employed in (15) is measured in m^2^ cycles/m and increases with the degradation of road smoothness class, following ISO 8608 [37,40]. Road roughness indices considered in the validation of the Kalman filter developed in this work are presented in Table 1, complete with the associated covariance matrix Q of the generated road profile height $z_{g}$. Use of the road profile as the disturbance input *w* has the advantage of linking disturbance covariance Q to parameters that can be inferred in real time during travel through accelerometers or with stereocameras [41,42]. In this way, corrections to Q could be performed in near real time, following its physical meaning of road profile irregularity. Such a physics-informed approach to covariance, where elements of the Q are updated based on the equations governing the system rather than through data-based estimations, offers significant improvements on state estimation while accelerating parameter tuning [43].

**Figure 3:**
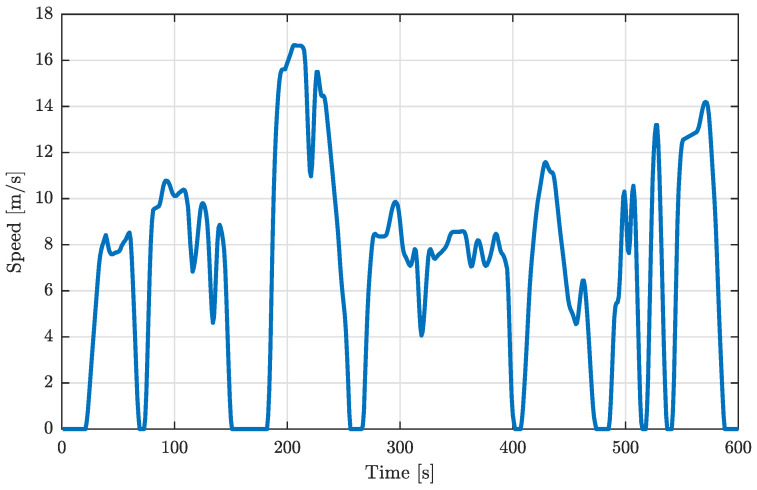
Speed profile imposed by the World Motorcycle Test Procedure.

Since the vertical road profile disturbance is heavily dependent on motorcycle speed *V*, it is necessary to assess the time history of such a variable throughout the duration of the simulation. To analyze the behavior of the Kalman filter over a cycle that is meaningful, realistic, and covers a wide range of speeds, the WMTC is employed, which is shown in Figure 3. The maximum velocity reached in the 599 s testing period is 16.67 m/s (60 km/h), with peak accelerations and decelerations of, respectively, 2.5 m/s^2^ and −2 m/s^2^.

For a rigorous validation of the Kalman filter’s performance in this application, true measurement data is required, complete with the associated noise level of employed sensors. Given the lack of this data in the literature and databases, the authors have chosen to add white noise *v* to the output of the state-space *y*, as shown in (2). Values of covariance are selected as 0.1 m^2^/s^4^ for the front unsprung mass vertical acceleration ${\ddot{z}}_{f}$ and 0.8 m^2^s/^4^ for the sprung mass vertical acceleration ${\ddot{z}}_{s}$, leading to errors of up to 1.25 m/s^2^ on the unsprung mass and 3.5 m/s^2^ on the sprung mass.

To properly assess the impact of the full-motorcycle model on the estimation of front fork travel speed, a baseline is required for comparison. Therefore, a second Kalman filter is defined based on a monocorner model, currently the most popular solution employed in [13]. To compare the results obtained with the two models, a performance metric is necessary, which analyzes the errors coming from each of the two models in the reconstruction of the front fork travel speed ${\hat{\dot{\xi }}}_{f}$. The function chosen to compare the performances is the ratio between the mean square error of the travel speed estimation and the mean square of ${\dot{\xi }}_{f}$ (the travel speed originating from the non-linear model) in (16), as done in the literature [13]:

(16) $$\eta_{KF}=\frac{\sum_{n=0}^{n_{steps}}{\left({\dot{\xi }}_{f}\left(n\right)-{\hat{\dot{\xi }}}_{f}\left(n\right)\right)}^{2}}{\sum_{n=0}^{n_{steps}}{\left({\dot{\xi }}_{f}\left(n\right)\right)}^{2}}.$$

## 3. State-Space Modelling

**Figure 4:**
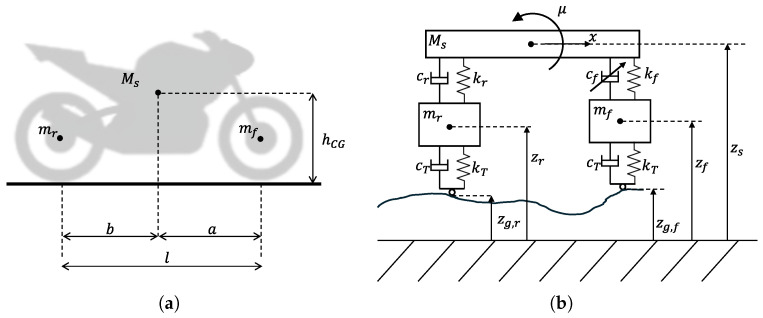
Representation of the vehicle model with the three masses and reference frames. The description of the vehicle parameters along with their values are collected in Table 2. (**a**) Correspondence between motorcycle elements and modelled masses. (**b**) Coordinate system of the full-motorcycle model.

The motorcycle analyzed in the study is represented as a system of three bodies: sprung mass *M*, front unsprung mass $m_{f}$ and rear unsprung mass $m_{r}$, as in Figure 4. Therefore, the system is characterized by four degrees of freedom: sprung mass vertical position $z_{s}$, vehicle pitch angle μ, front unsprung mass vertical position $z_{f}$, and rear unsprung mass vertical position $z_{r}$. Although simplified, such a system has been used for the design of a model predictive control (MPC) aimed at implementing a sky-hook control on both axles of a vehicle [44] and is commonly used to represent in-plane dynamics of motorcycles [5]. Geometrical and physical properties of the vehicle considered are reported in Table 2. Unsprung masses are lumped in the center of the wheel and their interface with the ground profile is modelled by a spring representing tire deformability, while its damping is small and generally neglected in [38].

A common state-space representation for the multi-body system of Figure 4b is the vector of vertical displacements and velocities shown in (17) [5,20].

(17) $$x={\left[z_{s},{\dot{z}}_{s},\mu ,\dot{\mu },z_{f},{\dot{z}}_{f},z_{r},{\dot{z}}_{r}\right]}^{T}$$

Velocities are required in the state-space formulation to express the equations in (2), starting from accelerations obtained from free-body diagrams.

The state space presented in (17), however, does not allow reconstruction of the telescopic fork travel speed using a Kalman filter. Therefore, a change of variables is required to include the telescopic fork travel speed as a state without increasing the system order [13]. The vertical positions of the sprung masses are replaced by their relative displacements with respect to the sprung-mass center of gravity, as in (18) and (19).

(18) $$\;\xi_{f}=z_{s}+a\sin\left(\mu \right)-z_{f}$$

(19) $$\xi_{r}=z_{s}-b\sin\left(\mu \right)-z_{r}$$

In the case of small pitch angles, it is possible to linearize the sine function with little to no impact on the results. Therefore, even though the vehicle model to be observed takes into consideration trigonometric functions, the state-space representation to be reconstructed by the Kalman filter substitutes the sine of the pitch angle μ with the angle itself, as in (20)–(25).

(20) $$\xi_{f}^{*}=z_{s}+a\mu -z_{f}$$

(21) $$\xi_{r}^{*}=z_{s}-b\mu -z_{r}$$

(22) $${\dot{\xi }}_{f}^{*}={\dot{z}}_{s}+a\dot{\mu }-{\dot{z}}_{f}$$

(23) $${\dot{\xi }}_{r}^{*}={\dot{z}}_{s}-b\dot{\mu }-{\dot{z}}_{r}$$

(24) $${\ddot{\xi }}_{f}^{*}={\ddot{z}}_{s}+a\ddot{\mu }-{\ddot{z}}_{f}$$

(25) $${\ddot{\xi }}_{r}^{*}={\ddot{z}}_{s}-b\ddot{\mu }-{\ddot{z}}_{r}$$

As a consequence, a linear time-invariant (LTI) state space with eight states $x^{*}$, two command inputs *u* and two disturbance inputs *w* is generated:

(26) $$x^{*}={\left[z_{s},{\dot{z}}_{s},\mu ,\dot{\mu },\xi_{f}^{*},{\dot{\xi }}_{f}^{*},\xi_{r}^{*},{\dot{\xi }}_{r}^{*}\right]}^{T}$$

(27) $$u={\left[F_{d},\dot{V}\right]}^{T}$$

(28) $$w={\left[z_{g,f},z_{g,r}\right]}^{T}.$$

To account for longitudinal acceleration-induced load transfer on the sprung and unsprung masses, the command input $\dot{V}$ is introduced via the corresponding tilting moment acting on the sprung mass, as computed in (29).

(29) $$M_{t}=M_{s}\left(h_{G}+z_{s}\right)\dot{V}$$

**Figure 5:**
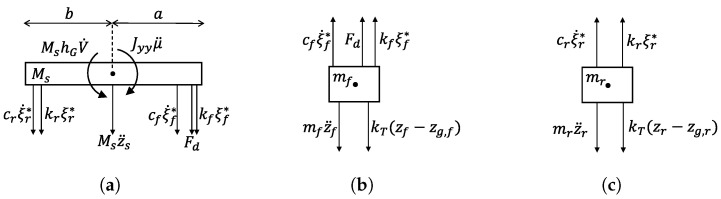
Free-body diagrams of the front and rear unsprung masses after linearization. (**a**) Free-body diagram of the sprung mass with the linearized tilting torque. (**b**) Free-body diagram of the front unsprung mass. (**c**) Free-body diagram of the rear unsprung mass.

To obtain a linear system, the input $\dot{V}$ must be decoupled from the state variable $z_{s}$. Accordingly, in the non-linear vehicle model, the tilting moment is computed as in (29), whereas in the linearized state space its dependence on the vertical position of the sprung mass $z_{s}$ is neglected, as shown in Figure 5a.

To evaluate the impact of linearization, a model verification is conducted between the LTI and the non-linear systems. This is performed computing the root mean square error (RMSE) between each state $x^{*}$ of the LTI system (26) and the corresponding state *x* of the non-linear system (17). To be able to compare the impact of linearization on each state, the value of RMSE is then normalized on the mean absolute value $\bar{x}$ of the non-linear state, as shown in (30).

(30) $$rRMSE=\frac{\sqrt{\frac{\sum_{n=0}^{n_{steps}}{\left(x^{*}\left(n\right)-x\left(n\right)\right)}^{2}}{n_{steps}}}}{\bar{x}}$$

**Figure 6:**
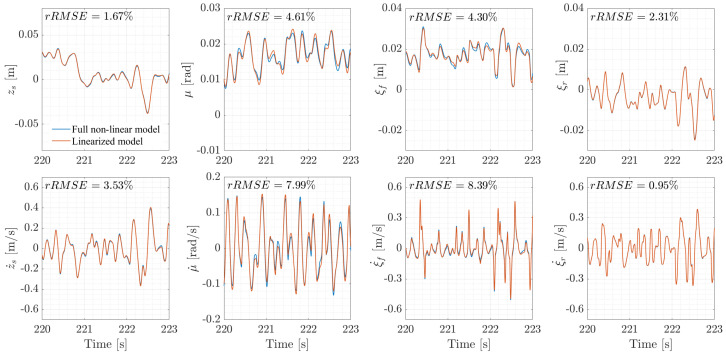
Comparison of the state spaces of the full non-linear and linearized models over the 3 s interval with the largest discrepancies between *x* and $x^{*}$. Road profile class C.

Since values of rRMSE below 10% are considered satisfactory [45], Figure 6 shows that the linearization did not introduce significant distortion to the state space, as the more affected state ${\dot{\xi }}_{f}$ has a value of rRMSE equal to 8.39%. The comparison is shown for a reduced time window of the driving cycle to better illustrate the differences between the two models. The time window was selected within a range of 220 and 223 s of the WMTC cycle, as it is the phase of the simulation where *x* and $x^{*}$ differ the most, due to the sharp speed changes imposed by the WMTC profile.

The state-space $x^{*}$ contains the four degrees of freedom of the system and the respective derivatives. To define matrices A and B, it is sufficient to derive the corresponding four acceleration equations coming from the free-body diagrams for the three masses. Since tire damping is neglected, only the the road profile $z_{g}$ must be taken into account [38]. A possible solution to also consider tire damping through the computation of ${\dot{z}}_{g,f}$ and ${\dot{z}}_{g,r}$ is to increase the state dimension from eight to ten by including $z_{g,f}$ and $z_{g,r}$ as state variables, shown in (31).

(31) $$x_{10}^{*}={\left[z_{s},{\dot{z}}_{s},\mu ,\dot{\mu },\xi_{f}^{*},{\dot{\xi }}_{f}^{*},\xi_{r}^{*},{\dot{\xi }}_{r}^{*},z_{g,f},z_{g,r}\right]}^{T}$$

To do so, a new disturbance input vector $w_{10}$ is defined, not constituted by the road profile as in (28), but by its derivatives, as in (33), a solution employed in the field of road profile estimation [46].

(32) $$u_{10}={\left[F_{d},\dot{V}\right]}^{T}$$

(33) $$w_{10}={\left[{\dot{z}}_{g,f},{\dot{z}}_{g,r}\right]}^{T}$$

(34) $${\dot{x}}_{10}^{*}\left(9\right)=w_{10}\left(1\right)$$

(35) $${\dot{x}}_{10}^{*}\left(10\right)=w_{10}\left(2\right)$$

However, the last two state variables depend purely on disturbance inputs and not on the state itself, as shown in (34) and (35). This means that the last two rows of the A matrix are nil and A is not at full rank, i.e., two of its eigenvalues are zeros. As a consequence, the observability matrix is not at full rank with the employed set of measurements and therefore the ten-state representation is not observable [47,48]. Such a property implies that the $x_{10}^{*}$ state space is not suitable for the definition of the system, as the Kalman filter requires observability of the state to be reconstructed [14,49].

As a consequence of $x_{10}^{*}$ un-observability, the Kalman filter is built around the linearized state space $x^{*}$, neglecting in the computation of A and B matrices the contribution of $\dot{w}$, which is accounted for in the non-linear motorcycle model that needs to be observed but has no relevance for the definition of the Kalman gain matrix $K_{k}$.

From the free-body diagram of the sprung mass $M_{s}$ shown in Figure 5a, it is possible to identify the derivatives of states $x^{*}\left(2\right)$ and $x^{*}\left(4\right)$, which are vertical acceleration ${\ddot{z}}_{s}$ and pitching acceleration $\ddot{\mu }$, presented in (36) and (37).

(36) $$\begin{array}{c} {\dot{x}}^{*}\left(2\right)=-\frac{k_{f}}{M_{s}}x^{*}\left(5\right)-\frac{c_{f}}{M_{s}}x^{*}\left(6\right)-\frac{k_{r}}{M_{s}}x^{*}\left(7\right)-\\ \frac{c_{r}}{M_{s}}x^{*}\left(8\right)-\frac{1}{M_{s}}u\left(1\right) \end{array}$$

(37) $$\begin{array}{c} {\dot{x}}^{*}\left(4\right)=-\frac{ak_{f}}{J_{yy}}x^{*}\left(5\right)-\frac{ac_{f}}{J_{yy}}x^{*}\left(6\right)+\frac{bk_{r}}{J_{yy}}x^{*}\left(7\right)+\\ \frac{bc_{r}}{J_{yy}}x^{*}\left(8\right)-\frac{a}{J_{yy}}u\left(1\right)+\frac{M_{s}h_{G}}{J_{yy}}u\left(2\right) \end{array}$$

To obtain the derivatives of states $x^{*}\left(6\right)$ and $x^{*}\left(8\right)$, the vertical accelerations of the front and rear unsprung masses ${\ddot{z}}_{f}$ and ${\ddot{z}}_{r}$ must first be obtained, as specified in (24) and (25). The resulting acceleration for the front unsprung mass, derived from the free-body diagram of Figure 5b, is presented in (38).

(38) $$\begin{array}{c} {\ddot{z}}_{f}=-\frac{k_{T}}{m_{f}}\left(z_{f}-w\left(1\right)\right)+\frac{k_{f}}{m_{f}}x^{*}\left(5\right)+\frac{c_{f}}{m_{f}}x^{*}\left(6\right)+\\ \frac{1}{m_{f}}u\left(1\right) \end{array}$$

The same can be done for rear unsprung mass $m_{r}$, whose free-body diagram is shown in Figure 5c, leading to (39).

(39) $$\begin{array}{c} {\ddot{z}}_{r}=-\frac{k_{T}}{m_{f}}\left(z_{r}-w\left(2\right)\right)+\frac{k_{r}}{m_{r}}x^{*}\left(7\right)+\frac{c_{r}}{m_{r}}x^{*}\left(8\right) \end{array}$$

Substituting the analytical forms of (36)–(38) into (24), it is therefore possible to obtain the state-space equation for the definition of ${\dot{x}}^{*}\left(6\right)$, shown in (40).

(40) $$\begin{array}{c} {\dot{x}}^{*}\left(6\right)=\frac{k_{T}}{m_{f}}x^{*}\left(1\right)+\frac{k_{T}a}{m_{f}}x^{*}\left(3\right)-\\ \left(k_{f}\left(\frac{1}{M_{s}}+\frac{a^{2}}{J_{yy}}\right)+\frac{k_{T}+k_{f}}{m_{f}}\right)x^{*}\left(5\right)-\\ \left(c_{f}\left(\frac{1}{M_{s}}+\frac{a^{2}}{J_{yy}}\right)+\frac{c_{f}}{m_{f}}\right)x^{*}\left(6\right)+\\ k_{r}\left(-\frac{1}{M_{s}}+\frac{ab}{J_{yy}}\right)x^{*}\left(7\right)+c_{r}\left(-\frac{1}{M_{s}}+\frac{ab}{J_{yy}}\right)x^{*}\left(8\right)-\\ \left(\frac{1}{M_{s}}+\frac{a^{2}}{J_{yy}}+\frac{1}{m_{f}}\right)u\left(1\right)+\frac{aM_{s}h_{G}}{J_{yy}}u\left(2\right)-\frac{k_{T}}{m_{f}}w\left(1\right) \end{array}$$

Analogously, substituting the analytical forms of (36), (37) and (39) into (25), the state-space equation for the definition of ${\dot{x}}^{*}\left(8\right)$ is obtained, shown in (41).

(41) $$\begin{array}{c} {\dot{x}}^{*}\left(8\right)=\frac{k_{T}}{m_{r}}x^{*}\left(1\right)-\frac{k_{T}b}{m_{r}}x^{*}\left(3\right)+\\ k_{f}\left(-\frac{1}{M_{s}}+\frac{ab}{J_{yy}}\right)x^{*}\left(5\right)+c_{f}\left(-\frac{1}{M_{s}}+\frac{ab}{J_{yy}}\right)x^{*}\left(6\right)-\\ \left(k_{r}\left(\frac{1}{M_{s}}+\frac{b^{2}}{J_{yy}}\right)+\frac{k_{T}+k_{r}}{m_{r}}\right)x^{*}\left(7\right)-\\ \left(c_{r}\left(\frac{1}{M_{s}}+\frac{b^{2}}{J_{yy}}\right)+\frac{c_{r}}{m_{r}}\right)x^{*}\left(8\right)+\\ \left(-\frac{1}{M_{s}}+\frac{ab}{J_{yy}}\right)u\left(1\right)-\frac{bM_{s}h_{G}}{J_{yy}}u\left(2\right)-\frac{k_{T}}{m_{r}}w\left(2\right) \end{array}$$

Having defined the system equations and the vehicle parameters, the four natural frequencies of the state space can be analytically computed as follows:

$$\begin{array}{cc} f_{heave} & =\frac{1}{2\pi }\sqrt{\frac{k_{f}+k_{r}}{M_{s}}}=2.18\;\mathrm{Hz};\\ f_{pitch} & =\frac{1}{2\pi }\sqrt{\frac{k_{f}a^{2}+k_{r}b^{2}}{J_{yy}}}=2.63\;\mathrm{Hz};\\ f_{unsprung,f} & =\frac{1}{2\pi }\sqrt{\frac{k_{f}+k_{T}}{m_{f}}}=21.09\;\mathrm{Hz};\\ f_{unsprung,r} & =\frac{1}{2\pi }\sqrt{\frac{k_{r}+k_{T}}{m_{r}}}=26.81\;\mathrm{Hz}. \end{array}$$

However, the natural frequencies for pitch and heave are too close to be decoupled and their relative mode shapes interact, as stated in the literature [17], highlighting the need for a full-motorcycle model to construct the state space. The frequencies obtained from the eigenvalues of the undamped state transition matrix are the following:

$$\begin{array}{cc} f_{I} & =1.86\;\mathrm{Hz};\\ f_{II} & =2.52\;\mathrm{Hz};\\ f_{III} & =21.05\;\mathrm{Hz};\\ f_{IV} & =26.75\;\mathrm{Hz}. \end{array}$$

where $f_{III}$ and $f_{IV}$ are the frequencies of two mode shapes dominated by unsprung mass vibration, while $f_{I}$ and $f_{II}$ are associated with mode shapes dominated by pitch and heave components.

Regarding the outputs of the described state space, the measured signals are the front unsprung mass vertical acceleration ${\ddot{z}}_{f}$ and the sprung mass vertical acceleration ${\ddot{z}}_{s}$, as done in the current literature [13]. With the same method, output matrices *C*, *D* and *H* are defined through the solution of free body diagrams of the masses, yielding (42) and (43).

(42) $$\begin{array}{c} y\left(1\right)={\ddot{z}}_{f}=-\frac{k_{T}}{m_{f}}\left(z_{f}-w\left(1\right)\right)+\frac{k_{f}}{m_{f}}x^{*}\left(5\right)+\\ +\frac{c_{f}}{m_{f}}x^{*}\left(6\right)+\frac{1}{m_{f}}u\left(1\right) \end{array}$$

(43) $$\begin{array}{c} y\left(2\right)={\ddot{z}}_{s}={\dot{x}}^{*}\left(2\right)=-\frac{k_{f}}{M_{s}}x^{*}\left(5\right)-\frac{c_{f}}{M_{s}}x^{*}\left(6\right)-\\ \frac{k_{r}}{M_{s}}x^{*}\left(7\right)-\frac{c_{r}}{M_{s}}x^{*}\left(8\right)-\frac{1}{M_{s}}u\left(1\right) \end{array}$$

**Figure 7:**
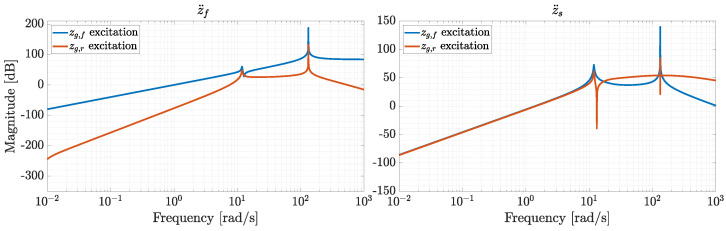
Frequency response to input disturbances $z_{g,f}$ and $z_{g,r}$ in magnitude for system outputs ${\ddot{z}}_{f}$ and ${\ddot{z}}_{s}$.

A frequency analysis of the resulting LTI system is presented in Figure 7, showing the magnitudes of the transfer functions from disturbance input *w* to the output *y*, represented by vertical accelerations measured on the front unsprung mass $\left({\ddot{z}}_{f}\right)$ and on the sprung mass $\left({\ddot{z}}_{s}\right)$, consistent with [13].

As the force actuated by the front damper $F_{d}$ is represented as a system input in Equations (36)–(41), the value of $c_{f}$ is considered to be zero. This yields two peaks at frequencies close to $f_{I}$ and $f_{III}$, which are noticeable in Figure 7, while $f_{IV}$ is suppressed by $c_{r}=3000$ Ns/m, making the corresponding mode shape overdamped. In frequency-domain analysis, it is common practice to consider in-phase and counter-phase excitations rather than single excitations, as shown in Figure 7. In particular, studies on suspension systems often analyze the frequency response of pitch and heave motions under in-phase and counter-phase road profile excitations [50,51]. In-phase excitation refers to the response of the vehicle sprung mass to road displacement inputs applied in the same direction at the front and rear tires, whereas counter-phase excitation refers to the response obtained when the disturbances are applied with a phase difference of 180°. When the road displacement $z_{g}$ is applied to the front and rear tires for phase $w_{\varphi }$ and counter-phase $w_{\bar{\varphi }}$ excitation, the inputs can be represented by the following vectors:

(44) $$\begin{array}{c} w_{\varphi }={\left[z_{g},z_{g}\right]}^{T},\;w_{\bar{\varphi }}={\left[z_{g},-z_{g}\right]}^{T}. \end{array}$$

**Figure 8:**
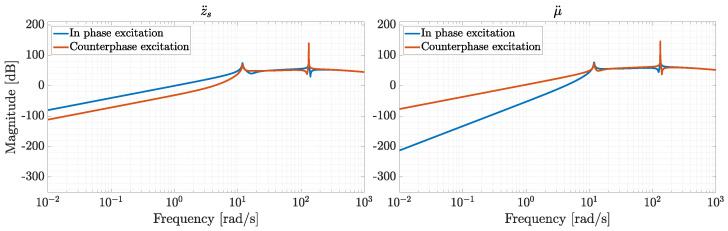
Frequency response to input disturbances $z_{g,f}$ and $z_{g,r}$ for phase ($z_{g,f}=z_{g,r}$) and counter-phase ($z_{g,f}=-z_{g,r}$) excitation for system outputs ${\ddot{z}}_{f}$ and ${\ddot{z}}_{s}$.

**Figure 9:**
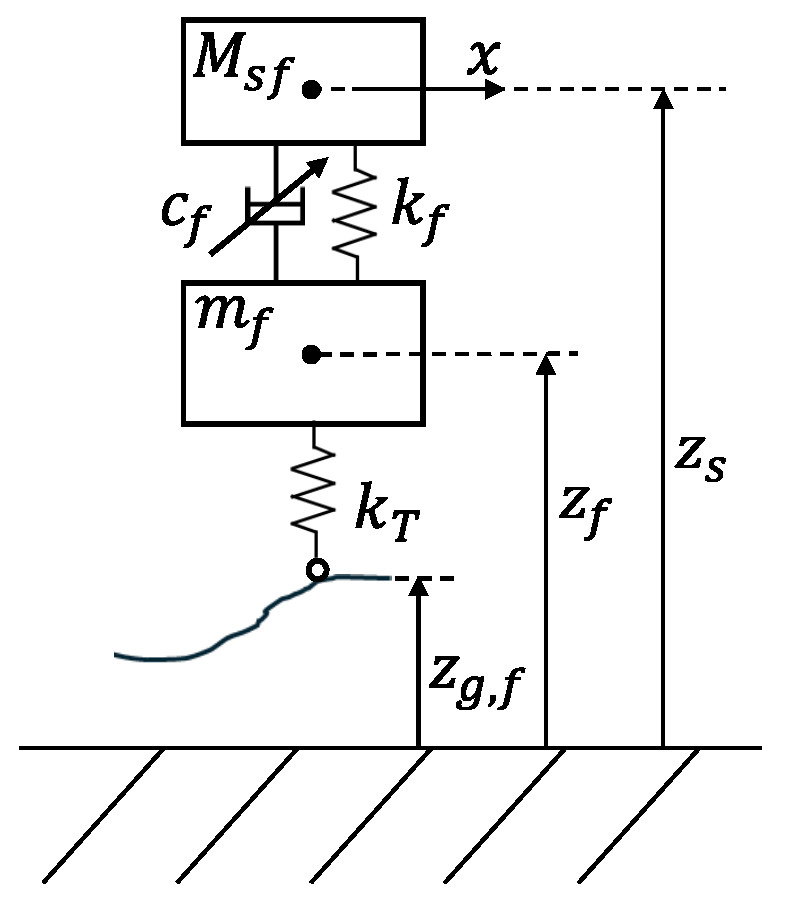
Representation of the monocorner model with the two masses and reference frames.

The results of phase and counter-phase excitation analysis are shown in Figure 8, where the same two resonance peaks as those of Figure 7 appear at 1.89 Hz and 21.04 Hz. The fact that these frequencies do not correspond to the theoretical peaks that would be obtained when analyzing each wheel on its own demonstrates the limitations of studying a monocorner model, shown in Figure 9.

The monocorner system forms the baseline of the study and is composed of only two masses, the front unsprung mass $m_{f}$ and the sprung mass $M_{sf}$, as done in [13]. In particular, since the state space represents only the front portion of the vehicle, the value of $M_{sf}$ is derived from the total sprung mass as in (45).

(45) $$\begin{array}{c} M_{sf}=M_{s}\frac{b}{l} \end{array}$$

Therefore, the state vector $x_{Q}$ is constituted by only four variables, as seen in (46), to account for the two degrees of freedom correlated to such a system, sprung mass vertical position $z_{s}$ and front unsprung mass vertical position $z_{f}$.

(46) $$\begin{array}{c} x_{Q}={\left[z_{s},{\dot{z}}_{s},z_{f},{\dot{z}}_{f}\right]}^{T} \end{array}$$

Such a model, neglecting the behavior of the rear unsprung mass, does not consider the disturbance input represented by the road profile below the rear wheel. Therefore, vector $w_{Q}$ is defined as in (47),

(47) $$\begin{array}{c} w_{Q}=z_{g,f} \end{array}$$

and the command input vector is reduced with respect to the full-motorcycle model, as the monocorner model cannot account for pitch μ. Thus, the effect of acceleration $\dot{V}$ disappears, and the command input $u_{Q}$ is defined as in (48).

(48) $$\begin{array}{c} u_{Q}=F_{d} \end{array}$$

To implement the Kalman filter in a real-world application, the continuous-time linear state space model reconstructed through matrix $K_{k}$, obtained by neglecting $\dot{w}$ in (31), must be converted into a discrete-time formulation, due to sampling effects in both inputs and measurements. Therefore, both the full-motorcycle-based and the monocorner-based state-space models are discretized prior to defining their respective observer matrices. Tustin discretization [52] is used, as it provides a convenient mapping from the continuous to the discrete domain. For both systems, the selected sample time $T_{s}$ is 1 ms.

## 4. Results and Discussion

Simulations of the motorcycle subjected to a randomly generated road profile are conducted to evaluate the improvements in travel speed estimation achieved by the full-motorcycle model relative to the monocorner model. Each simulation has a duration of 599 s, corresponding to the length of one WMTC cycle, during which the speed changes between 0 m/s and 16.7 m/s. A total of eight simulations are performed, one per each road classification according to ISO Standard 8608. The simulations aim to evaluate the performance of the two Kalman filters in reconstructing the telescopic fork travel speed ${\dot{\xi }}_{f}$. The metric defined in (16) is computed for each road profile and summarized in Table 3.

**Figure 10:**
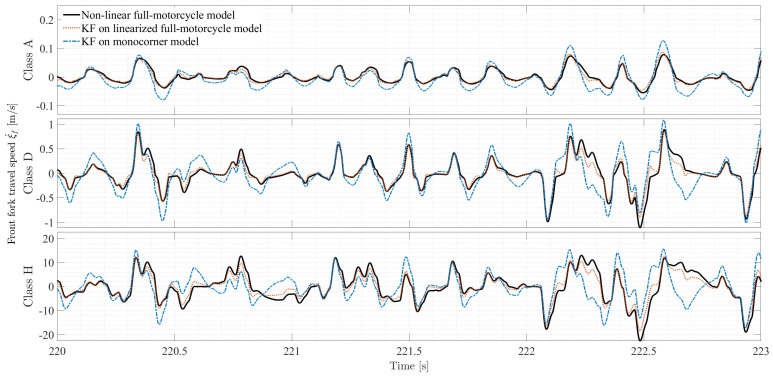
Front fork travel speed comparison of different Kalman filters against the non-linear full-motorcycle model over a 3 s interval for different road profile classes.

**Figure 11:**
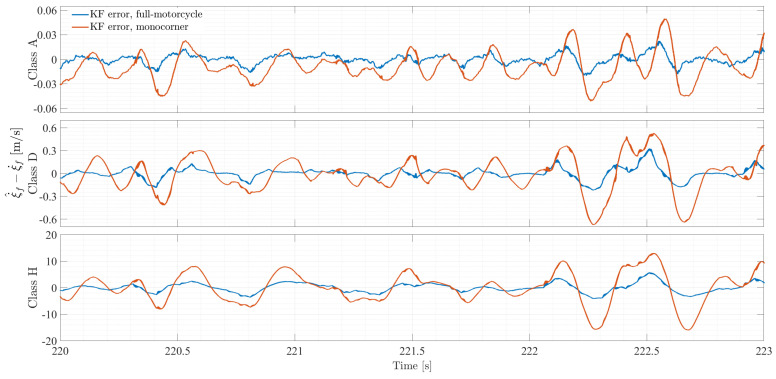
Estimation error comparison of Kalman filter-based full-motorcycle and monocorner model over a 3 s interval for different road profile classes.

Figure 10 compares the simulation results with their Kalman filter reconstruction for road classes A, D, and H (selected for brevity). By comparing the curves, it is evident how the implementation of a full-motorcycle model leads to a better estimation of the front fork travel speed ${\dot{\xi }}_{f}$, as its trace follows the full non-linear vehicle simulation data more closely than the trace related to the baseline filter. This statement is confirmed by the trend of the relative error for each of the road classes over the same time window, shown in Figure 11. The first panel of Figure 10 displays the performance of the two filters for the best road class for a time window of 3 s for clarity purposes. Both filters show good reconstruction of the front fork travel speed; however, the baseline displays multiple instances of signal underestimation and overestimation. The first panel of Figure 11 shows an error that is bounded between ±0.03 m/s for the full-motorcycle filter and ±0.06 m/s for the monocorner variant. Indeed, with reference to Table 3, the error reduction for this road class is shown to be 88.6%.

The advantages of using a Kalman filter based on a full-motorcycle model are more profound under harsher road classes. Considering a class D road profile (second panel in Figure 10), the full-motorcycle Kalman filter displays an error reading that is 91.3% lower compared to that of the monocorner model filter. As seen in the second panel of Figure 11, the error is shown to be bounded between ±0.3 m/s for the full-motorcycle filter and ±0.6 m/s for the baseline. With reference to class H road profile (third panel in Figure 10), the full-motorcycle Kalman filter exhibits a closer reproduction of the travel speed, with an error reading reduction of 92.5% compared to the baseline. The third panel in Figure 11 evidences an error that is bounded between ±4 m/s for the full-motorcycle filter and ±16 m/s for the monocorner variant within the time window selected for analysis.

The performance improvements of a Kalman filter designed with a full-motorcycle model in reconstructing the front fork have been shown for multiple road classes within this study. It must be noted, however, that the performance of the filter is executed in a simulation environment. White noise is added to the output of the non-linear state-space model such that the behavior of the two Kalman filters can be studied as close to reality as possible. The limitation, of course, is that the selected noise injected into the system in this study may not be truly representative of scenarios with different accelerometer types and their mountings on the vehicle. This has been aimed to be appropriately mitigated by imposing noise levels on the unsprung mass that are near those available in the literature [34], while a more conservative noise value is imposed on the sprung mass due to the presence of engine vibrations. These informed assumptions are deemed suitable for evaluating the performance of the full-motorcycle model Kalman filter with respect to a monocorner model filter. Subsequent work in this field is aimed at validating the results obtained in this study, considering the use of low-cost sensors.

Additionally, as the damper force estimation relies on the estimated value of the elongation speed, the importance of the accuracy of this variable is further highlighted. By keeping the LUT constant between both filters, part of the reduced estimation accuracy of the monocorner-based Kalman filter may be attributed to the reduced accuracy of the estimated force. Nevertheless, provided that the estimation of the damper force for such filtering techniques requires a high accuracy of the estimation of the elongation speed, which in turn dictates the accuracy of the estimation of the said variable in the following time instant, a higher-order model shows higher utility. These considerations, along with the improved performance shown, suggest that a higher-order model is recommended for the robust estimation of the motorcycle front fork elongation speed within a Kalman filtering approach.

## 5. Conclusions

This paper evaluates the performance of two Kalman filters designed to reconstruct the telescopic fork travel speed of a motorcycle. The analysis is based on the World Motorcycle Test Procedure and eight road profiles to evaluate the performance of a baseline defined using a monocorner model and a proposed solution based on a full-motorcycle model. The motorcycle is modelled through a state-space representation, where the plant includes non-linear effects, while the Kalman filter approximates these non-linearities and is seen to perform with satisfactory reconstruction of the front suspension travel speed. By incorporating the motorcycle acceleration along the driving cycle, the effect of vehicle pitch induced by inertial forces on the telescopic fork travel speed is included. However, greater improvements in the speed estimation are seen with respect to the effect of road irregularities. The full-motorcycle model is shown to outperform the monocorner model in reconstructing the target quantity, achieving a mean squared error reduction of 92.5% in the worst road class. With these considerations made, the future steps include the development of a higher-level control logic for a semi-active damper, evaluating its performance using the travel speed estimated by the two Kalman filters. In addition, implementation of these models on microprocessor platforms for real-time evaluation is provisioned.

## Figures and Tables

**Table 1:** Numeric properties of road profiles. $G_{v}$ taken as $c_{average}$ from [40].

Class	$G_{v}$ [m^2^ · Cycles/m]	*Q* Covariance [m^2^]
A	$1.60\times 10^{-7}$	$3.16\times 10^{-5}$
B	$6.40\times 10^{-7}$	$1.26\times 10^{-4}$
C	$2.56\times 10^{-6}$	$5.05\times 10^{-4}$
D	$1.02\times 10^{-5}$	$2.02\times 10^{-3}$
E	$4.10\times 10^{-5}$	$8.08\times 10^{-3}$
F	$1.64\times 10^{-4}$	$3.23\times 10^{-2}$
G	$6.55\times 10^{-4}$	$1.29\times 10^{-1}$
H	$2.62\times 10^{-3}$	$5.17\times 10^{-1}$

**Table 2:** Properties of the vehicle.

Symbol	Description	Numerical Value	Unit
*M*	Total vehicle mass including the driver	380	kg
$M_{s}$	Total sprung mass including the driver	360	kg
$J_{yy}$	Sprung mass pitching moment of inertia	110	kg·m^2^
$m_{f}$	Front unsprung mass	12	kg
$m_{r}$	Rear unsprung mass	8	kg
*l*	Wheelbase	1.320	m
*a*	Front-center of gravity distance	0.642	m
*b*	Rear-center of gravity distance	0.678	m
$h_{G}$	Center of gravity height	0.500	m
$k_{f}$	Front spring stiffness	25,777	N/m
$k_{r}$	Rear spring stiffness	42,000	N/m
$k_{T}$	Tire vertical stiffness	185,000	N/m
$c_{f}$	Passive front damping coefficient	0	Ns/m
$c_{r}$	Passive rear damping coefficient	3000	Ns/m

**Table 3:** Summary of results for different road profiles.

Class	Full-Motorcycle $\eta_{\mathrm{KF}}$ [%]	Monocorner $\eta_{\mathrm{KF}}$ [%]
A	6.54	57.46
B	4.50	63.31
C	3.83	64.49
D	4.93	56.61
E	6.43	69.25
F	7.13	81.01
G	7.21	86.83
H	7.07	94.47

## Data Availability

No new data were created or analyzed in this study. Data sharing is not applicable to this article.
